# Prognostic Significance of the Neutrophil-to-Lymphocyte Ratio in Primary Liver Cancer: A Meta-Analysis

**DOI:** 10.1371/journal.pone.0096072

**Published:** 2014-05-02

**Authors:** Tong-Chun Xue, Lan Zhang, Xiao-Yin Xie, Ning-Ling Ge, Li-Xin Li, Bo-Heng Zhang, Sheng-Long Ye, Zheng-Gang Ren

**Affiliations:** 1 Liver Cancer Institute, Zhongshan Hospital, Fudan University, Shanghai, P.R. China; 2 Key Laboratory of Carcinogenesis and Cancer Invasion (Fudan University), Ministry of Education, Shanghai, P.R. China; 3 Department of Medical Statistics, Zhongshan Hospital, Fudan University, Shanghai, P.R. China; University of Pisa, Italy

## Abstract

The neutrophil-to-lymphocyte ratio (NLR) is a useful biomarker that reflects systemic inflammation responses. However, the prognostic value of the NLR in patients with primary liver cancer (PLC) remains controversial. We performed a meta-analysis of 26 studies (comprising 4,461 patients) to evaluate the association between the pre-treatment NLR and clinical outcomes of overall survival (OS) and disease-free survival (DFS) in patients with PLC. The correlation between NLR and tumor characteristics or other inflammation-related parameters was also assessed. Data were synthesized using the random-effects model of DerSimonian and Laird, and the hazard ratio (HR) or odds ratio (OR) with 95% confidence interval (CI) was used to estimate effect size. Our analysis indicated that a high NLR predicted poor OS (HR, 2.102; 95% CI: 1.741–2.538) and DFS (HR, 2.474; 95% CI: 1.855–3.300) for PLC. A high NLR was associated with the presence of tumor vascular invasion (OR: 1.889, 95% CI: 1.487–2.400; *p*<0.001) and an elevated alpha-fetoprotein level (OR: 1.536; 95% CI: 1.152–2.048; *p* = 0.003). Thus, we conclude that a high NLR indicates a poor prognosis for patients with PLC and may also be predictive for PLC invasion and metastasis. Subgroup analysis suggested that the predictive role of NLR in cholangiocarcinoma is limited, and a further large study to confirm these findings is warranted.

## Introduction

Inflammatory responses have been shown to correlate closely with tumor progression, including promotion of angiogenesis and tumor invasion through the up-regulation of cytokines. The capacity of tumor cells to invade, access the vasculature, and metastasize is modulated by signals from the primary tumor microenvironment, bloodstream, and the new microenvironment (secondary site).[1] [2] Accumulating evidence suggests that these signals are correlated closely with inflammation-related cells, including neutrophils,[3], [4] platelets,[5] and lymphocytes. Neutrophils and lymphocytes in the primary tumor microenvironment are correlated closely with local inflammation and immune responses, respectively, and play prominent regulatory roles in tumor progression.[4]

The neutrophil-to-lymphocyte ratio (NLR) indicates the balance of the inflammatory and immune systems, making the NLR a useful index that reflects systemic inflammation responses. Evidence suggests that the presence of neutrophils in the tumor stroma is associated with a poor prognosis,[4], [6] whereas lymphocyte infiltration around a tumor has been reported to be associated with a better prognosis.[7], [8] Therefore, the NLR has attractive prognostic value for patients with tumors.[9]

Primary liver cancer (PLC) malignancies, primarily hepatocellular carcinoma (HCC) or intracholangiocarcinoma (ICC), occur worldwide with high mortality and morbidity. Accumulated evidence indicates that the progression of PLC is correlated closely with both inflammation[10] and immunocytes.[11] Recent studies suggest a potential prognostic role of the NLR in the treatment strategies of PLC, including hepatic resection,[12], [13], [14] liver transplantation,[15], [16], [17] radiofrequency ablation (RFA),[18], [19] and trans-arterial chemo-embolization (TACE)[20], [21]. However, most of these studies were composed of relatively small samples. Moreover, some studies had negative results.[22] [23] Therefore, whether NLR is a suitable prognostic factor for liver cancer remains controversial.

In recent years, significant advances have been made in liver cancer treatment. However, the appropriate stratification of cancer patients and subsequent allocation to surgical and palliative treatments remains a challenge. The conventional prognostic factors for PLC have limitations. For example, alpha-fetoprotein (AFP) is not suitable for predicting the prognosis of HCC patients with a normal AFP level or patients with ICC. In the present meta-analysis, we evaluated the prognostic roles of the NLR for survival following treatment of PLC. Our results show that a high NLR can strongly predict poor survival of PLC, indicating the predictive value of the NLR as a new biomarker in PLC.

## Materials and Methods

### Search strategy and selection criteria

We performed a search of Ovid Medline (1945 to present with daily update; in-process and other non-indexed citations), EMBASE (from 1974 to October 16, 2013), Web of Knowledge including SCIE (Science Citation Index Expanded), CPCI-S (Conference Proceedings Citation Index-Science) from 1997 to the present, and the Cochrane library up to October 2013. The main terms “hepatocellular carcinoma”, “liver cancer”, “hepatoma”, and “intracholangiocarcinoma” in the title as well as “neutrophil*” and “lymphocyte*” in the title/abstract, were used. References cited in the retrieved articles were also searched for relevant titles. This meta-analysis was conducted in accordance with the guidelines provided by the PRISMA statement (Checklist S1).

Studies concerning the prognostic role of the NLR in liver cancer, including HCC or ICC, were the first choice for inclusion. Further criteria for selection included data on overall survival (OS) or disease-free survival (DFS) for evaluation and pre-treatment determination of the NLR. Each included study was approved by an ethics committee or institutional review board. Exclusion criteria were: (1) no access to the full text for quality assessment and data extraction; (2) review articles; and (3) non-clinical studies or case reports.

### Data extraction and quality assessment

Three investigators independently reviewed all potentially eligible studies and collected data on patient and study characteristics. Discrepancies were resolved by discussion and consensus. The Newcastle-Ottawa Scale (NOS) was used to assess study quality. The NOS consists of three parameters of quality: selection (0–4 points), comparability (0–2 points), and outcome assessment (0–3 points). The maximum possible score is 9 points, representing the highest quality methodological study.

### Data synthesis and analysis

The OS and DFS were assessed as the primary measures of the treatment effect using the hazard ratio (HR) with 95% confidence interval (CI). The methods for incorporating summary time-to-event data into the meta-analysis were described previously.[24] In addition to the survival analysis, the relationship between the NLR and clinical-pathologic characteristics was also assessed.

Pooled analyses were performed using DerSimonian-Laird random-effects models. Sensitivity analyses were performed to determine the stability of the overall treatment effects. We excluded one study at a time to ensure that no single study would be solely responsible for the significance of any result. Accumulated analyses were used to evaluate the total trend of the studies. Statistical heterogeneity was measured using I-squared statistics. Subgroup analysis and meta-regression analyses were conducted to explore and explain the diversity (heterogeneity) among the results of different studies. Publication bias was assessed using Begg's funnel plot and Egger's test. All *p* values were two-tailed, and statistical significance was set at 0.05. Statistical analyses were performed using STATA version 12.0 (StataCorp, College Station, TX, USA) and Comprehensive Meta Analysis version 2.0 (Biostat, Inc., Englewood, NJ, USA).

## Results

### Included eligible studies

**Figure 1:**
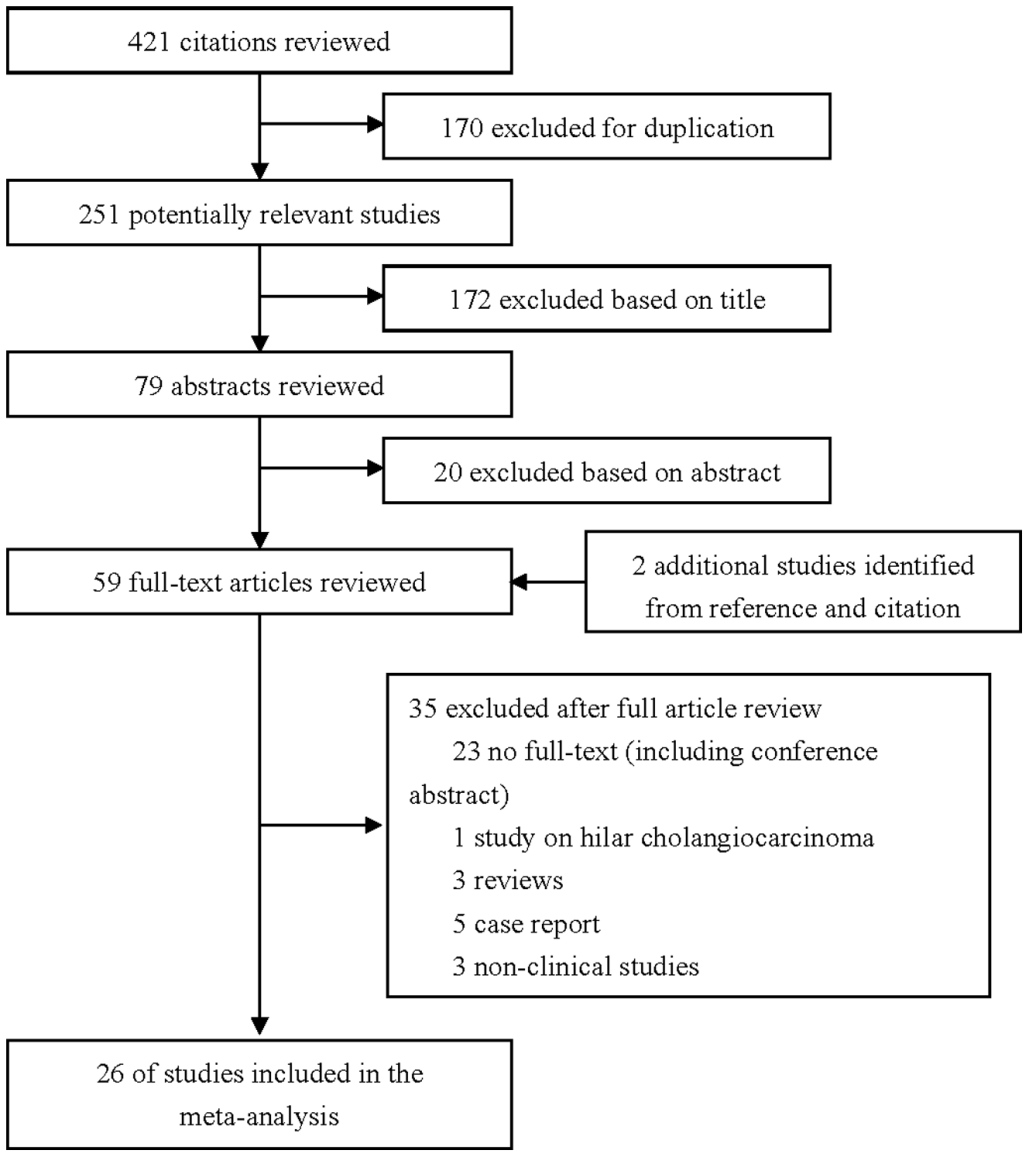
Search flow diagram for studies included in the meta-analysis.

The flow chart of the study selection for the meta-analysis is shown in Figure 1. Briefly, of the initial 421 hits, 61 articles were retrieved for detailed evaluation and 26 studies [12]–[38] satisfying the inclusion criteria were finally analyzed. Ten trials were from Western countries, including four studies from the United States, two studies from the United Kingdom, three studies from Italy, and one study from Brazil. Sixteen studies were from Eastern countries, including 10 from China, 5 from Japan, and one from South Korea. Because of the lack of available full text, conference abstracts were not included. One study of hilar cholangiocarcinoma was also excluded.[39] Quality assessment of the trials is shown in Table 1.

**Table 1:** Baseline characteristics of the twenty-six studies included in the meta-analysis.

Study	Year	Country	NOS	Treatment	Cancer Type	Sample Size	Mean Age(y)	Men(%)	Sample	High NLR Definition	Number of patients with “high” NLR	Survival Analysis	Hazard Ratios	Follow-up (median, mo)
Gomez	2008	UK	6	Resection	ICC	26	55	8(31%)	Peripheral Blood	NLR> = 5	12	OS/DFS	Estimated	23
Gomez(2)	2008	UK	7	Curative Resection	HCC	96	65	72(75%)	Peripheral Blood	NLR> = 5	26	OS/DFS	Reported in text	30
Halazun	2009	US	6	Transplantation	HCC	150	57	119(79.3%)	Peripheral Blood	NLR> = 5	13	OS/DFS	Reported in text	37
Guo	2009	CN	6	Curative Resection	HCC	91	31	76(83.5%)	Peripheral Blood	NLR> = 2	49	OS/DFS	Reported in text	NP
Li	2011	CN	6	Curative Resection	HCC	197	NP	165(83.8%)	Cancer tissue	CD66b/CD8> = 90%	178	OS/DFS	Estimated	29
Huang	2011	CN	5	TACE	HCC	145	49	80(55.2%)	Peripheral Blood	NLR> = 3.3	59	OS	Reported in text	10
Chen	2011	CN	6	Radiofrequency ablation	HCC	158	66	95(60.1%)	Peripheral Blood	NLR> = 2.4	81	OS/DFS	Reported in text	34
Wang	2011	CN	6	Transplantation	HCC	101	48	92(91.1%)	Peripheral Blood	NLR> = 3	33	OS/DFS	Reported in text	34
Bertuzzo	2011	IT	6	Transplantation	HCC	219	57	186(84.9%)	Peripheral Blood	NLR> = 5	23	OS/DFS	Reported in text	40
Wang(2)	2011	CN	5	Transplantation	HCC	76	48	71(93%)	Peripheral Blood	NLR> = 2.5	37	DFS	Reported in text	35
Kinoshita	2012	JP	6	Multiple Treatments	HCC	150	72	106(70.7%)	Peripheral Blood	NLR> = 5	15	OS	Estimated	18
Pinato	2012	UK & IT	6	Multiple Treatments	HCC	112	65	90(80.4%)	Peripheral Blood	NLR> = 5	25	OS	Reported in text	NP
Pinato(2)	2012	UK & IT	6	TACE	HCC	54	63	40(74.1%)	Peripheral Blood	NLR> = 5	9	OS	Reported in text	NP
Li	2012	CN	5	Curative Resection	HCC	82	58	68(82.9%)	Peripheral Blood	NLR> = 5	15	OS/DFS	Estimated	48
McNally	2013	US	6	TACE	HCC	104	61	76(73.1%)	Peripheral Blood	NLR> = 5	18	OS	Estimated	NP
Mano	2013	JP	6	Resection	HCC	958	67	689(71.9%)	Peripheral Blood	NLR> = 2.81	238	OS/DFS	Estimated	NP
Oh	2013	KR	7	TACE	HCC	318	58	240(75.5%)	Peripheral Blood	NLR> = 2.3	189	OS	Reported in text	13.9
Yoshizumi	2013	JP	6	Transplantation	HCC	104	58	63(60.6%)	Peripheral Blood	NLR> = 4	21	DFS	Reported in text	NP
Limaye	2013	US	7	Transplantation	HCC	160	55	130(81.3%)	Peripheral Blood	NLR> = 5	28	OS/DFS	Reported in text	38
Motomura	2013	JP	8	Transplantation	HCC	158	57	92(58.2%)	Peripheral Blood	NLR> = 4	26	OS/DFS	Estimated	40
Sullivan	2013	US	6	Multiple Treatments	HCC*	75	61	57(76%)	Peripheral Blood	NLR> = 5	NP	OS	Reported in text	12
Fu	2013	CN	7	Curative Resection	HCC	282	51	249(88.3%)	Peripheral Blood	NLR> = 2	147	OS/DFS	Reported in text	29
Dan	2013	CN	6	Radiofrequency ablation	HCC	178	57	159(89.3%)	Peripheral Blood	NLR> = 1.9	68	OS/DFS	Reported in text	53
Lai	2013	BE	6	Transplantation	HCC	146	58	116(79.5%)	Peripheral Blood	NLR> = 5.4	30	Intent-to-treat survival/DFS	Estimated	NP
Harimoto	2013	JP	6	Transplantation	HCC	167	NP	NP	Peripheral Blood	NLR> = 4	26	survival after recurrence	Estimated	NP
Li	2013	CN	5	TACE	HCC	154	50	134(87%)	Peripheral Blood	NLR> = 2.5	69	OS	Estimated	15

*, included 3 ICC; NLR, neutrophil-to-lymphocyte ratio; NOS, Newcastle–Ottawa Scale; NP, not reported; OS, overall survival; DFS, disease-free survival.

The main features of the 26 studies included in the meta-analysis are shown in Table 1. All of the studies were retrospective cohort studies. All were reported within the past 5 years, and 50% were reported in 2013. The studies included 4,461 patients, 1,732 of whom received hepatic resection, 336 received RFA treatment, 1,281 received liver transplantation, and 775 received TACE treatment. Most of the studies concerned HCC; only one study concerned ICC. Three study centers each reported two studies; however, all the studies were assessed in the final meta-analysis because they involved different cancer types, treatments, or NLR cut-off values. The percentage of men ranged from 31% to 93%, and the mean age ranged from 31 to 72 years. HRs were estimated for each study using the available data or methods described above. Each individual study reported a “high” NLR level with survival data; the NLR cut-off value was determined using different methods among studies.

### NLR and survival (OS and DFS)

#### Pooled HR

**Figure 2:**
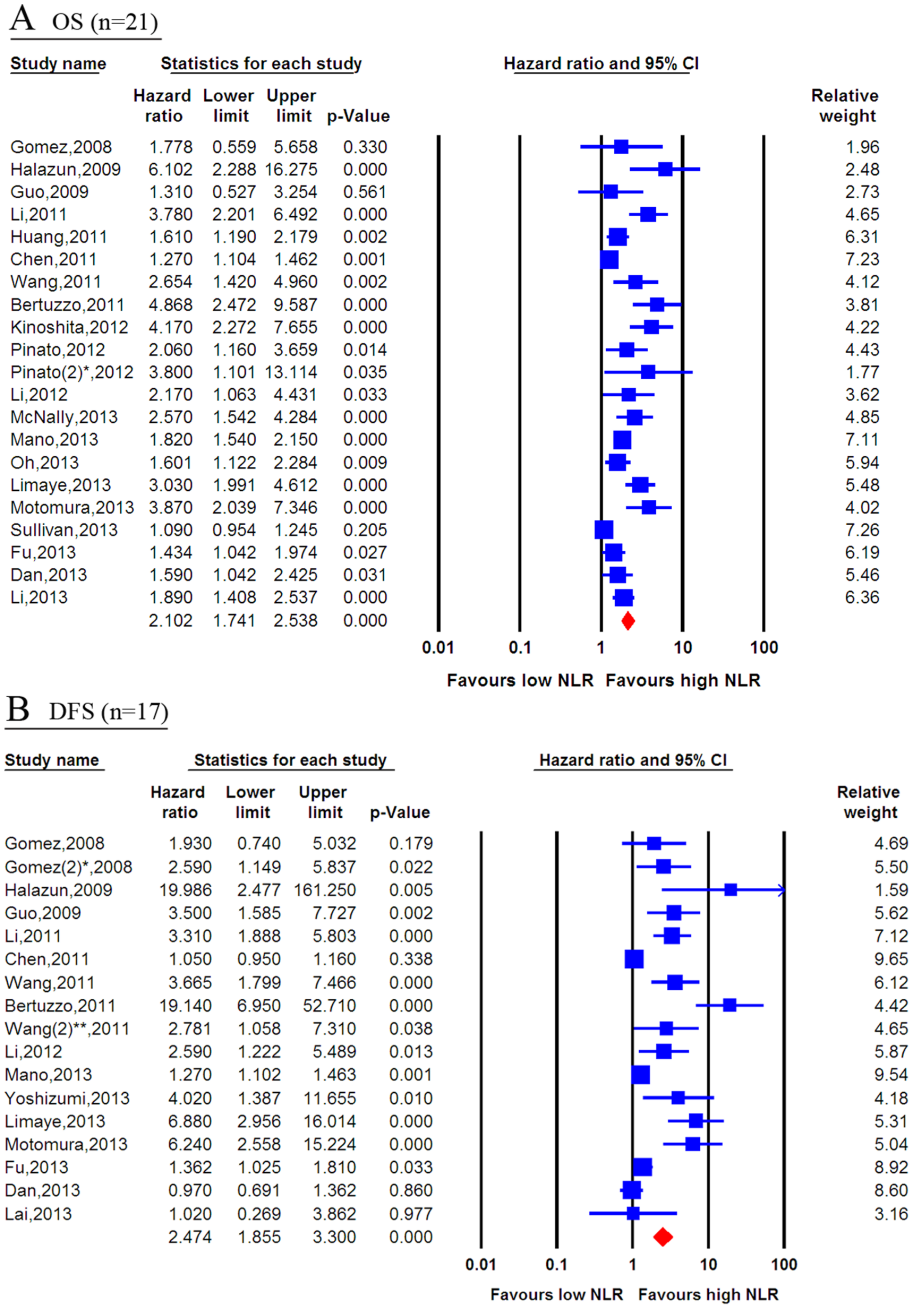
Forest plots of the association between the NLR and survival of patients with primary liver cancer. A random-effects model was used. (A) Forest plot of the association between the NLR and OS of 21 studies. (B) Forest plot of the association between the NLR and DFS of 17 studies. Blue represents the HR estimate of each study, whereas red represents the overall pooled effective size. NLR  =  neutrophil-to-lymphocyte ratio; OS  =  overall survival; DFS  =  disease-free survival; *, the different study by Gomez; **, the different study by Wang.

Twenty-one studies provided information regarding OS. The prognostic role of a high NLR for OS is shown in Figure 2A. The data indicate high statistical heterogeneity with I-squared value of 81.3% (*p*<0.001) and the 95% CIs for the results of individual trials had a wide range. The prognostic role of a high NLR in survival was favored in most studies, except for three that had no significant results. The pooled estimate of the high NLR was significant (HR, 2.102; 95% CI: 1.741, 2.538; *p*<0.001), indicating that patients with a high baseline NLR had poor OS. Additionally, 17 studies provided data concerning DFS. As shown in Figure 2B, the pooled estimate of the high NLR for DFS was significant (HR, 2.474; 95% CI: 1.855, 3.300; *p*<0.001), indicating that patients with high pre-treatment NLR had poor DFS.

Sensitivity analyses suggested that the pooled effect of the NLR on OS was not affected by sequential exclusion of each study (Fig. S1). Moreover, accumulated analyses indicated that the pooled trend of the HR for the NLR regarding OS was not affected following sequential inclusion of each study (Fig. S2). Similar to the OS, sensitivity analyses suggested that the pooled effect of NLR on DFS was not affected following sequential exclusion of each study in turn (data not shown).

#### Sub-group analyses

**Figure 3:**
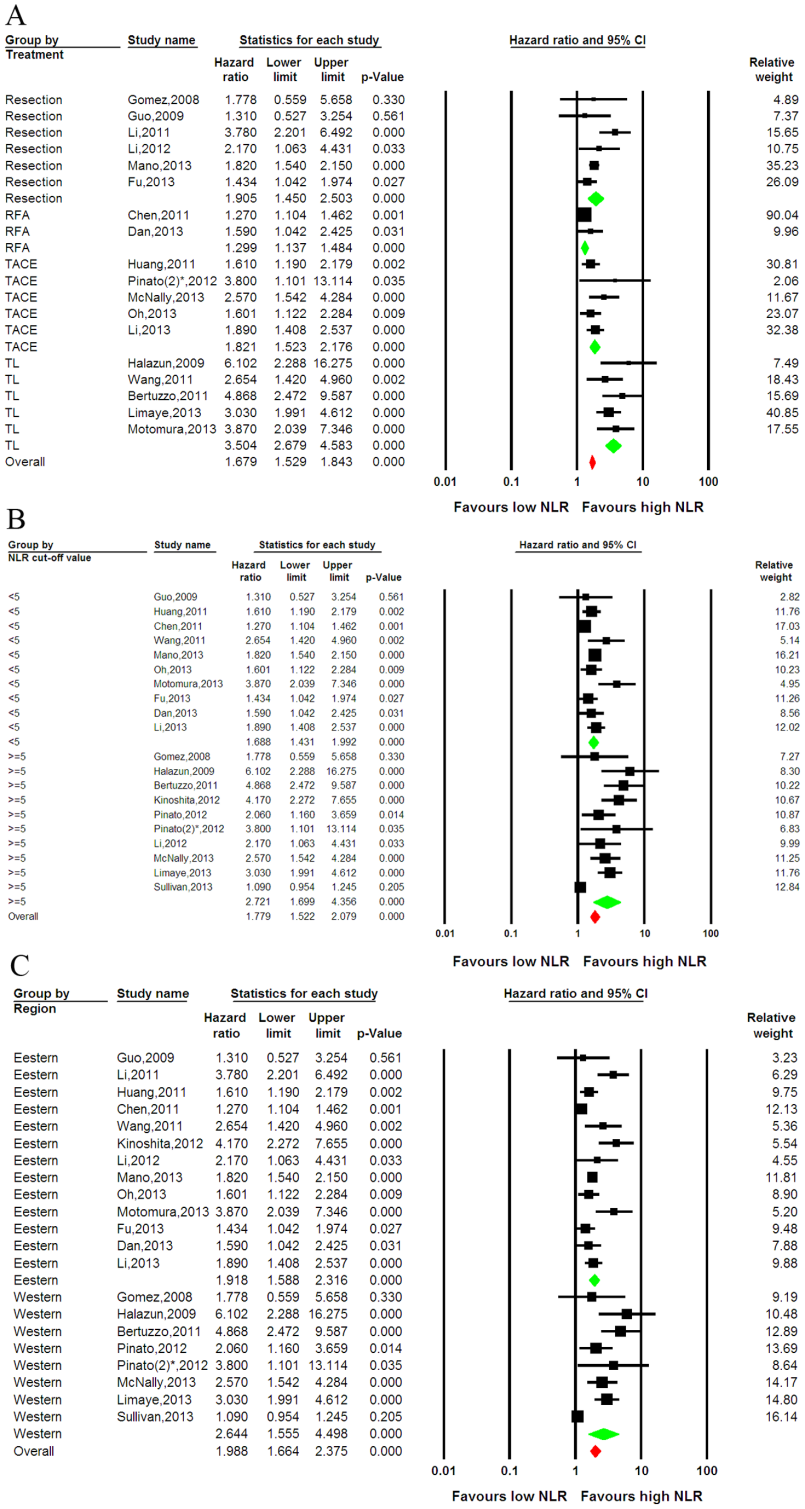
Stratified forest plots of the association between the NLR and OS. (A) Subgroup analysis in patients who received different treatments. (B) Subgroup analysis in studies with an NLR cut-off value less than or greater than 5. (C) Subgroup analysis was based on the region in which the study was reported, including 13 Eastern studies and 8 Western studies. Green represents the subgroup pooled effective size, whereas red represents the overall pooled effective size. NLR  =  neutrophil-to-lymphocyte ratio; OS  =  overall survival; CI  =  confidence interval; TL  =  transplantation; *, the different study by Pinato.

We further explored potential causes of the heterogeneity in the meta-analysis. First, we analyzed the significance of a high NLR with respect to the OS for patients who received different treatments. Among six resection studies, two RFA studies, five TACE studies, and five liver transplantation studies, the prognostic role of the NLR in OS was favored in most studies and the pooled estimate of the NLR was significant (HR, 1.679; 95% CI: 1.529, 1.843; *p*<0.001) (Fig. 3A). Statistical heterogeneity was found in the subgroups that received multiple treatments (I-squared = 90.72%; *p*<0.001) and resection (I-squared = 49.4%, *p* = 0.078); heterogeneity was not found in the subgroups that received RFA (I-squared = 0.0%; *p* = 0.322), TACE (I-squared = 6.97%, *p* = 0.367), or transplantation (I-squared = 0.0%; *p* = 0.487).

Because the NLR cut-off values were clearly different among the studies, ranging from 1.9 to 5.0, we performed subgroup analysis based on the NLR cut-off value. In 10 studies with an NLR cut-off less than 5 and 10 studies with an NLR greater than 5, the prognostic role of NLR in OS was favored in most studies, and the pooled estimate of the NLR was significant (HR, 1.779; 95% CI: 1.522, 2.079; *p*<0.001) (Fig. 3B). Statistical heterogeneity was found in subgroups with an NLR greater than 5 (I-squared = 87.4%; *p*<0.0001 and in subgroups with NLR <5 (I-squared = 62.6%; *p* = 0.004).

Because patients with HCC in Western or Eastern countries have a different medical background with respect to liver disease, sub-group analyses based on region were also performed. Although two studies from the Western group and one study from the Eastern group suggested a negligible role of the NLR in OS, the prognostic role of the NLR in OS was favored in most studies and the pooled estimate of the NLR was significant (HR, 1.988; 95% CI: 1.664, 2.375; *p*<0.001) (Fig. 3C). Statistical heterogeneity was noted (I-squared = 59.4%, *p* = 0.031) in both the Eastern (I-squared = 72.7%; *p* = 0.001) and Western (I-squared = 87.9%; *p*<0.001) groups.

Of all the 26 studies, only one clearly reported the prognostic role of the NLR in OS for ICC. Consequently, sub-group analysis suggested that the pooled estimate of the NLR regarding OS in ICC patients was not significant (*p* = 0.330). In contrast, the pooled estimate of the NLR regarding HCC was very strong (HR, 2.203; 95% CI: 1.828, 2.655; *p*<0.001) (Fig. S3A). In addition, sub-group analyses suggested a prognostic role of the NLR in OS based on the median follow-up time (Fig. S3B) or sampling method (Fig. S3C).

Regarding DFS, sub-group analyses was also performed based on treatment, NLR cut-off value, region, cancer type, follow-up time, and sampling method, as shown in Figure S4. The pooled results were similar to those for OS except that the NLR had no prognostic role in the DFS of the subgroup with RFA (HR, 1.043; 95% CI: 0.948, 1.148; *p* = 0.385).

#### Meta-regression analyses

**Figure 4:**
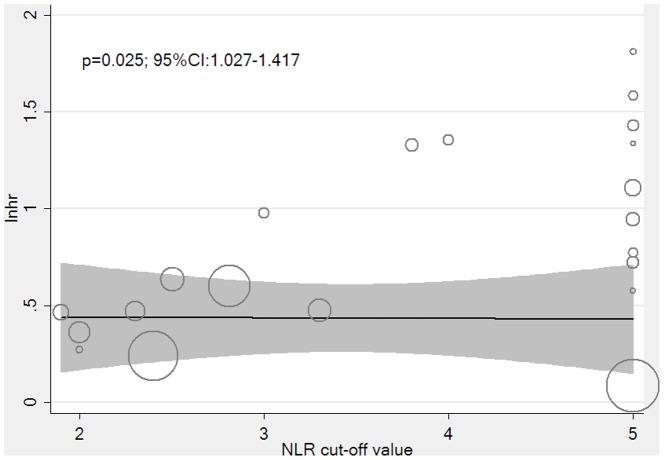
Meta-regression between the NLR cut-off value and lnhr. NLR  =  neutrophil-to-lymphocyte ratio; lnhr  =  log of the hazard ratio.

Meta-regression analysis was also used to explore the heterogeneity in the studies for OS. Twenty-one studies were analyzed, and 10 features—publication year, sample size, mean age, proportion of men, number of high NLR values, research region, treatment type, cancer type, sampling method, and NLR cut-off value—were examined.[6], [7], [9]–[13] Data concerning the median follow-up time could not be analyzed because they were incomplete. The adjusted R-squared value from univariate meta-regression suggested that the NLR cut-off value could explain 18.85% heterogeneity alone (*p* = 0.025, 95% CI: 1.027, 1.417) (Fig. 4), whereas cancer type could potentially explain another 18.85% heterogeneity alone (*p* = 0.143). Other factors had no significant role in heterogeneity because they had adjusted R-squared values of less than 5%. Additionally, the adjusted R-squared values suggested that the NLR cut-off value (*p* = 0.001, 95% CI: 1.119, 1.431) and cancer type (*p*<0.001, 95% CI: 0.265, 0.628) combined could explain 84.46% heterogeneity, indicating that the NLR cut-off value and cancer type were the main sources of heterogeneity.

### NLR and clinicopathologic characteristics

#### NLR and vascular invasion

**Figure 5:**
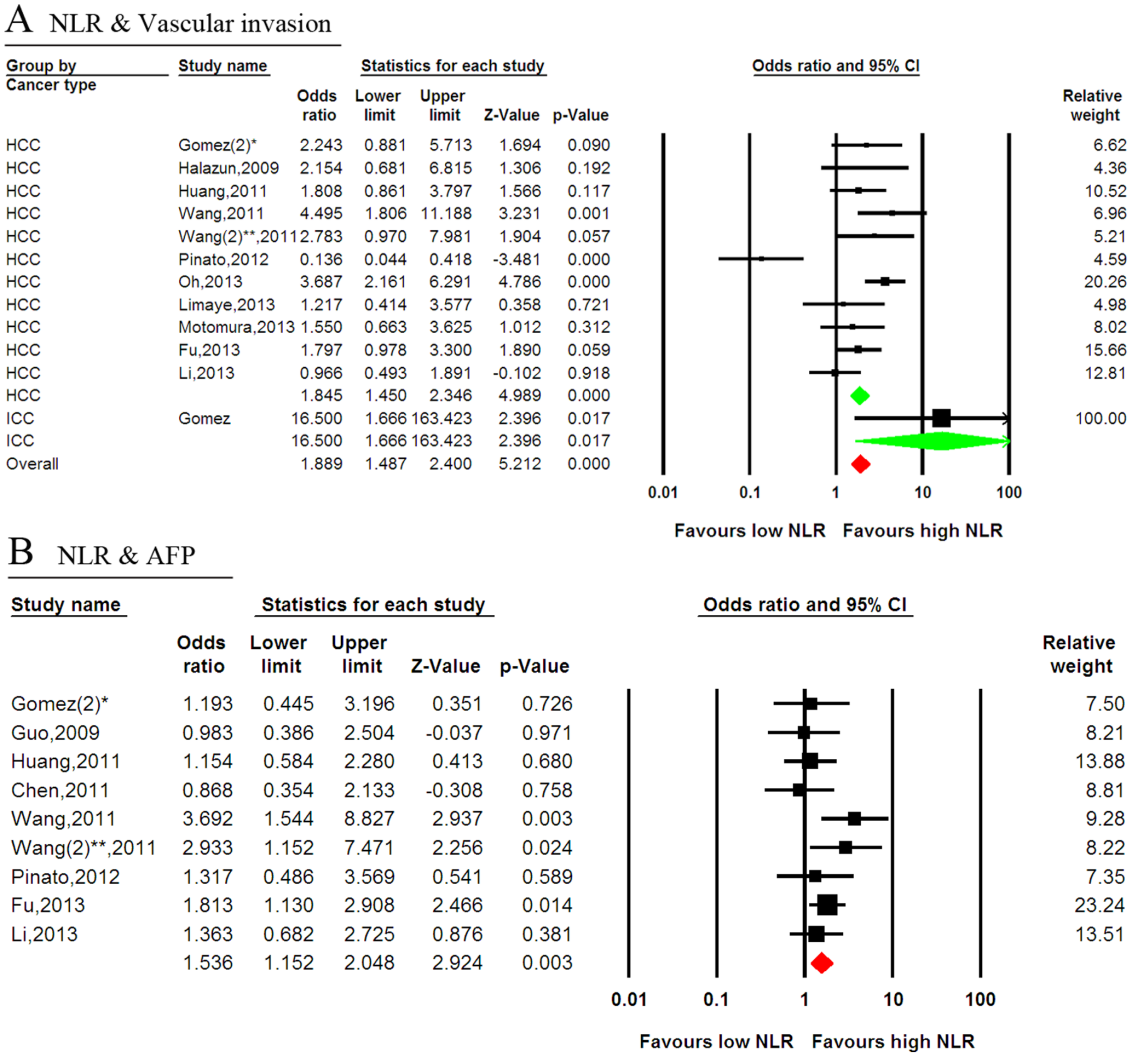
Forest plots of the association between the NLR and tumor characteristics. (A) The association between the NLR and vascular invasion. (B) The association between the NLR and an elevated AFP level. Green represents the subgroup pooled effective size, whereas red represents the overall pooled effective size. NLR  =  neutrophil-to-lymphocyte ratio; AFP  =  alpha feto-protein; CI  =  confidence interval; *, the different study by Gomez; **, the different study by Wang.

Twelve studies reported data concerning high NLR and tumor vascular invasion in PLC, including 11 HCC studies and one ICC study. A high NLR seemed to be associated with the presence of vascular invasion in most studies, and statistical significance was observed in two studies. Conversely, one study demonstrated a correlation between a low NLR and vascular invasion. Combined data from all 12 studies showed a trend for a correlation between a high NLR and vascular invasion (odds ratio (OR): 1.889; 95% CI: 1.487–2.400; *p*<0.001), although heterogeneity was evident in the sub-group with HCC (I-squared = 87.3%; *p*<0.001). Sub-group analysis indicated that a high NLR was associated with vascular invasion in both HCC and ICC (Fig. 5A).

#### NLR and AFP

In addition to its diagnostic role, AFP has been suggested to be a prognostic factor for the survival of patients who received various treatments. Nine studies reported data on high NLR and AFP in PLC. Of these, six suggested no correlation between a high NLR and an elevated AFP level and three reported statistical significance. Combined data from all nine studies showed a correlation between high NLR and an elevated AFP level (OR: 1.536; 95% CI: 1.152–2.048; *p* = 0.003) (Fig. 5B).

#### NLR and CRP or PLR

C-reactive protein (CRP), a marker of acute inflammation, and the platelet-to-lymphocyte ratio (PLR) are both potentially useful prognostic factors for survival in PLC. Of 26 included studies, five[15], [25], [30], [31], [33] reported the prognostic role of CRP in PLC. Four studies[25], [30], [31], [33] confirmed the prognostic value of CRP; one study[31] showed that the combined use of CRP and NLR provided incremental prognostic information, and one study[30] suggested a strong correlation between CRP and NLR. Only two studies[25], [33] reported the prognostic role of PLR in PLC.

#### NLR and differentiation or extra-hepatic spread

Seven studies reported data on high NLR values and poorly differentiated cells; however, only one study suggested a potential correlation between high NLR and poor differentiation. Additionally, combined data could not support a correlation (OR: 1.279; 95% CI: 0.911–1.797; *p* = 0.155; Fig. S5A). Four studies reported the potential relationship between NLR and extra-hepatic spread. Although one study suggested that a high NLR correlated with extra-hepatic spread, the pooled data did not show statistical significance (OR: 1.570; 95% CI: 0.706–3.491; *p* = 0.268; Fig. S5B).

### Publication bias

The results of Egger's test suggested evidence for publication bias regarding the DFS studies (*p*<0.001). However, OS publication bias was not obvious (Fig. S6).

## Discussion

Inflammation, as a protective response, has been shown to play critical roles in tumor development. Inflammation-related neutrophils and immunocytes participate in communication between the microenvironment and tumor cells. However, these cell types play discrepant roles in the systemic inflammation response. The NLR potentially balances the functions of neutrophils and immunocytes, making it a useful prognostic factor. Although accumulated evidence has suggested the prognostic value of the NLR in PLC, controversial results persist. The present meta-analysis is the first to show that a high NLR shows prominent prognostic significance regarding the survival of patients with PLC, particularly those with HCC. A prognostic role was demonstrated for both OS and DFS of patients with PLC. Similar to our study, a recent meta-analysis confirmed the prognostic value of the NLR in colorectal cancer.[40]

Regarding liver cancer, some tumor characteristics have been shown to have prognostic value, including vascular invasion[41] and an increased AFP level[42]. The current meta-analysis showed that a high NLR correlated closely with vascular invasion and an elevated AFP level, both of which are known to be correlated with invasion of tumor cells and to be prognostic factors for poor survival of patients. Similar to the NLR as a prognostic factor related to inflammation, CRP, the Glasgow Prognostic Score[43] and the PLR have also been shown to be potential prognostic factors. Previous studies have suggested that an elevated CRP concentration is associated with poor survival in patients with HCC.[44] Moreover, platelets have been shown to induce an epithelial-mesenchymal-like transition[45] and platelet-derived nucleotides were shown to promote tumor cell transendothelial migration and metastasis.[5] Therefore, the PLR should also be investigated as a potential prognostic factor. Further research is required to compare the prognostic roles of these inflammation-based indices in PLC.

Only one study[27] in the present meta-analysis used the CD66b+/CD8+ ratio instead of the NLR, and the prognostic role of CD66b+/CD8+ in the current study seem to be stronger than that in other studies according to subgroup analysis. It has been shown that CD4+ T cells and CD8+ T cells play discrepant, and even opposing roles, in the tumor microenvironment. CD8+ lymphocytes promote the killing of tumor cells, whereas regulatory CD4+ T cells potentially promote the progression of liver cancer. Moreover, inflammation can promote tumor invasion and metastasis through the recruitment of regulatory T lymphocytes. Therefore, the neutrophil-to-CD8+ T cell ratio[46] may have stronger prognostic value for survival than the conventional NLR index, a finding that requires future confirmation. In addition, the prognostic role of osteopontin in PLC suggested by one meta-analysis has been confirmed, mainly on the basis of immunohistochemical staining methods.[47] Therefore, the combination of osteopontin and the NLR, a value that considers the microenvironment for both local and systemic effects, may have a stronger prognostic value in PLC.

Subgroup estimation in the present study indicated that a high NLR was an effective prognostic factor for poor survival for patients who received various types of treatment, including radical resection, RFA, transplantation, and non-radical TACE. Although the predictive role of NLR for pooled OS of patients who received RFA was positive, the pooled DFS showed a negative predictive role of NLR in these patients, in part because of the few RFA studies and discrepancies among studies. Recently, another study that was published after our search period for the current meta-analysis suggested the prognostic value of the NLR for patients who received sorafenib monotherapy.[48] Although liver cancer has an uneven worldwide distribution, sub-group analysis based on region suggested that the prognostic roles of NLR were in accordance with either the Eastern or Western region. The main cause of heterogeneity in that study may lie in the subgroup analysis based on the NLR cut-off value, treatment type, and study region. Conversely, the heterogeneity of the present meta-regression analysis was mainly due to the NLR cut-off value, particularly combined with cancer type. Some studies set the NLR cut-off based on the receiver operating characteristic method, whereas others set the cut-off by referencing previous evidence. However, subgroup analysis confirmed that both NLR less than 5 and NLR greater than 5 were effective prognostic factors, in accordance with the screening results in some studies. In addition, the difference in the follow-up period between radical therapy and non-radical treatment may affect heterogeneity. Furthermore, studies concerning transplantation indicated that patients always received other treatments before transplantation.

### Study limitations

Although we comprehensively evaluated the association between the NLR and tumor outcome, several limitations of the current meta-analysis should be acknowledged. Drawbacks pertinent to the present meta-analysis were mainly differences in characteristics among the included studies. Heterogeneity is a potential problem that may affect the interpretation of the results of all meta-analyses. Subgroup and meta-regression suggested that cancer type might partially explain the heterogeneity. Of the 26 included studies, only one concerned treatment for ICC. Therefore, the pooled predictive role of high NLR cut-off for survival of ICC patients in subgroup analysis was negative because of the single negative report in the Gomez study. The limited sample size and number of ICC studies induced heterogeneity in this meta-analysis, and larger studies of ICC are required to provide further evidence. In addition, the NLR cut-off value was set differently among studies, which was one of the main sources of heterogeneity. Another weakness of our study was publication bias, which was evident in the meta-analysis. This might be because positive results were more likely to be published than negative ones. A tendency for journals to only publish positive results leads to a larger magnitude of an association in pooled analysis than the actual value.

In conclusion, this study demonstrated that the NLR is associated with poor survival of PLC. The NLR is a useful biomarker that provides essential information for predicting survival and tumor invasiveness of patients with PLC patients who received treatment. Subgroup analysis suggested that the predictive role of NLR in ICC is limited, and a further large study to confirm these findings is warranted.

## Supporting Information

Figure S1 **Sensitivity analyses of the association between the NLR and overall survival.** The analyses were carried out by the sequential exclusion of each study in turn. NLR  =  neutrophil-to-lymphocyte ratio; CI  =  confidence interval; *, the different study by Pinato.
(TIF)

Figure S2 **Accumulated analysis of the association between the NLR and overall survival**. The analyses were carried out by the sequential addition of each study. NLR  =  neutrophil-to-lymphocyte ratio; CI  =  confidence interval; *, the different study by Pinato.
(TIF)

Figure S3 **Stratified forest plots of the association between the NLR and OS.** (A) Subgroup analysis was based on cancer type. (B) Subgroup analysis in studies with a median follow-up time less than or more than 3 years. (C) Subgroup analysis was based on the sampling method. Green represents the subgroup pooled effective size, whereas red represents the overall pooled effective size. NLR  =  neutrophil-to-lymphocyte ratio; OS  =  overall survival; CI  =  confidence interval; *, the different study by Pinato.
(TIF)

Figure S4 **Stratified forest plots of the association between the NLR and DFS.** (A) Subgroup analysis in patients who received different treatments. (B) Subgroup analysis in studies with an NLR cut-off value less than or greater than 5. (C) Subgroup analysis was based on the region in which the study was reported. (D) Subgroup analysis was based on cancer type. (E) Subgroup analysis in studies with a median follow-up time less than or greater than 3 years. (F) Subgroup analysis was based on the sampling method. Green represents the subgroup pooled effective size, whereas red represents the overall pooled effective size. NLR  =  neutrophil-to-lymphocyte ratio; DFS  =  disease-free survival; CI  =  confidence interval; *, the different study by Gomez; **, the different study by Wang.
(TIF)

Figure S5 **Forest plots of the association between the NLR and tumor characteristics.** (A) The association between the NLR and differentiation of tumor cells. (B) The association between the NLR and extra-hepatic spread. Green represents the subgroup pooled effective size, whereas red represents the overall pooled effective size. NLR  =  neutrophil-to-lymphocyte ratio; CI  =  confidence interval; **, the different study by Wang.
(TIF)

Figure S6 **Funnel plots for the hazard ratios of recessive modes in the included studies.** (A) OS (n = 26). (B) DFS (n = 17).
(TIF)

Checklist S1 **PRISMA checklist of the meta-analysis.**
(DOC)
