# Supplementary figures and images for: Prognostic Significance of the Neutrophil-to-Lymphocyte Ratio in Primary Liver Cancer: A Meta-Analysis
Source: PLoS One. 2014 May 2;9(5):e96072. doi: 10.1371/journal.pone.0096072 (PMC4008563; doi:10.1371/journal.pone.0096072)

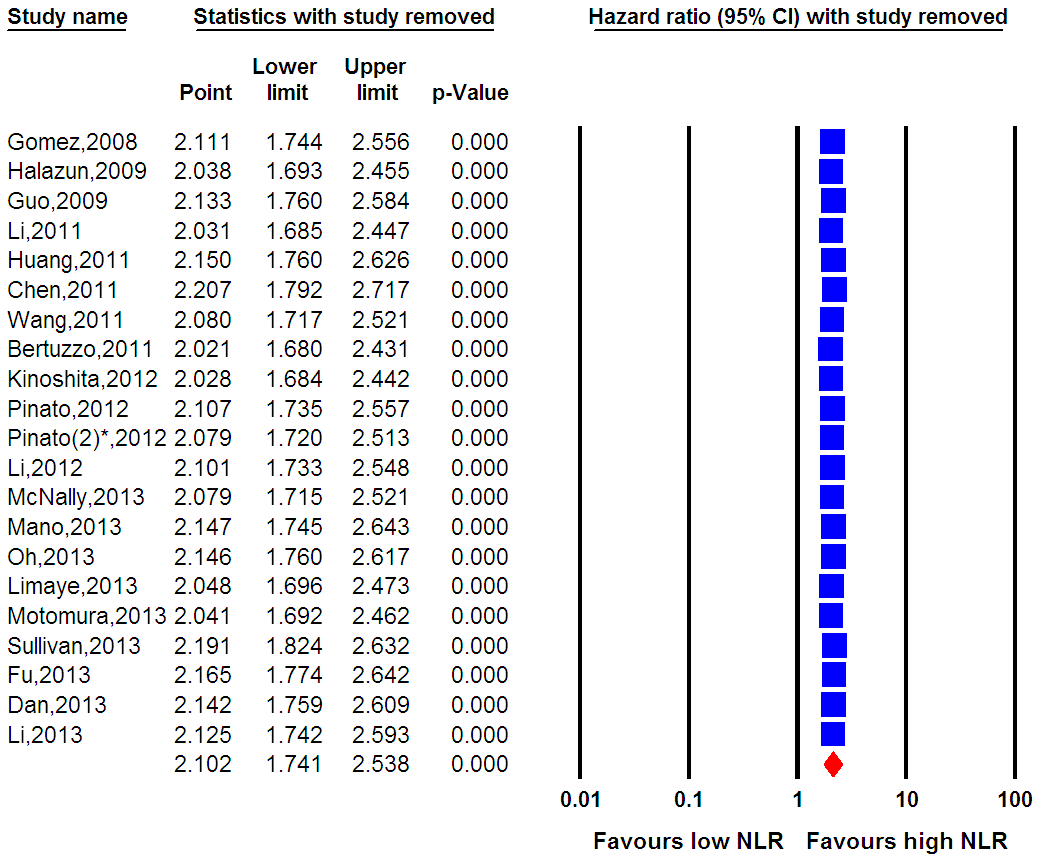

Supplement: Figure S1 — Sensitivity analyses of the association between the NLR and overall survival. The analyses were carried out by the sequential exclusion of each study in turn. NLR = neutrophil-to-lymphocyte ratio; CI = confidence interval; *, the different study by Pinato. (TIF) [file pone.0096072.s001.tif]

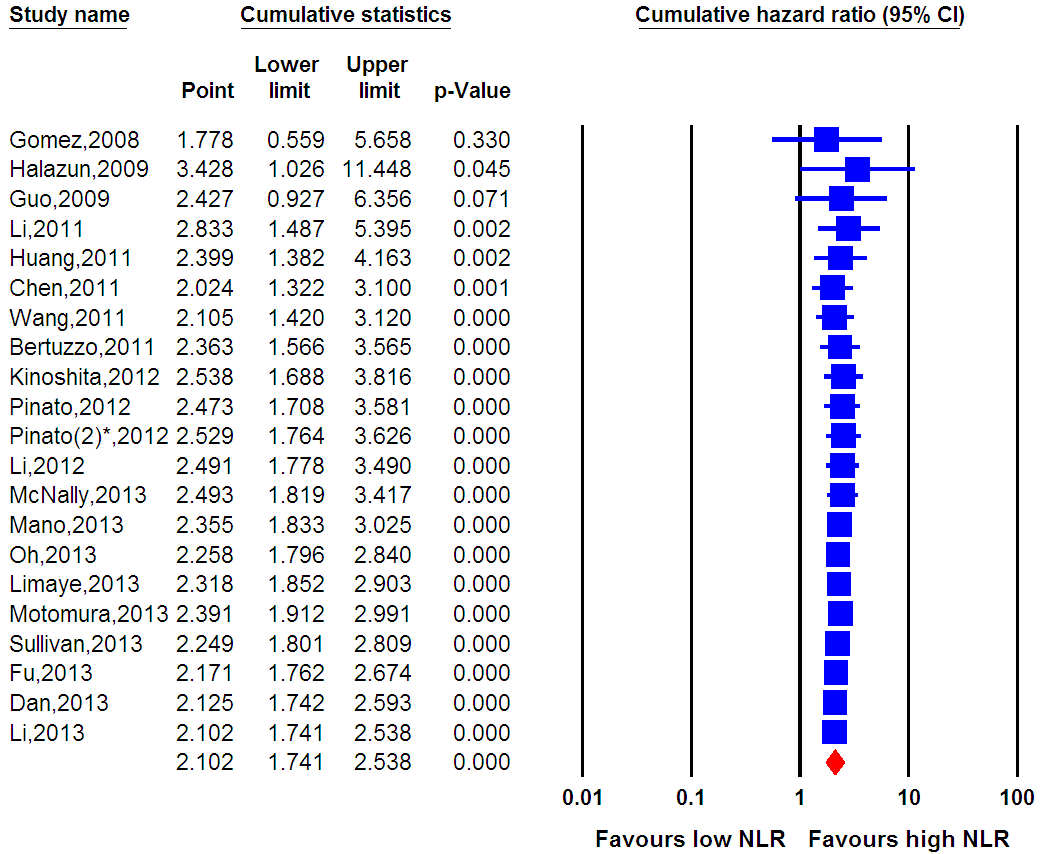

Supplement: Figure S2 — Accumulated analysis of the association between the NLR and overall survival. The analyses were carried out by the sequential addition of each study. NLR = neutrophil-to-lymphocyte ratio; CI = confidence interval; *, the different study by Pinato. (TIF) [file pone.0096072.s002.tif]

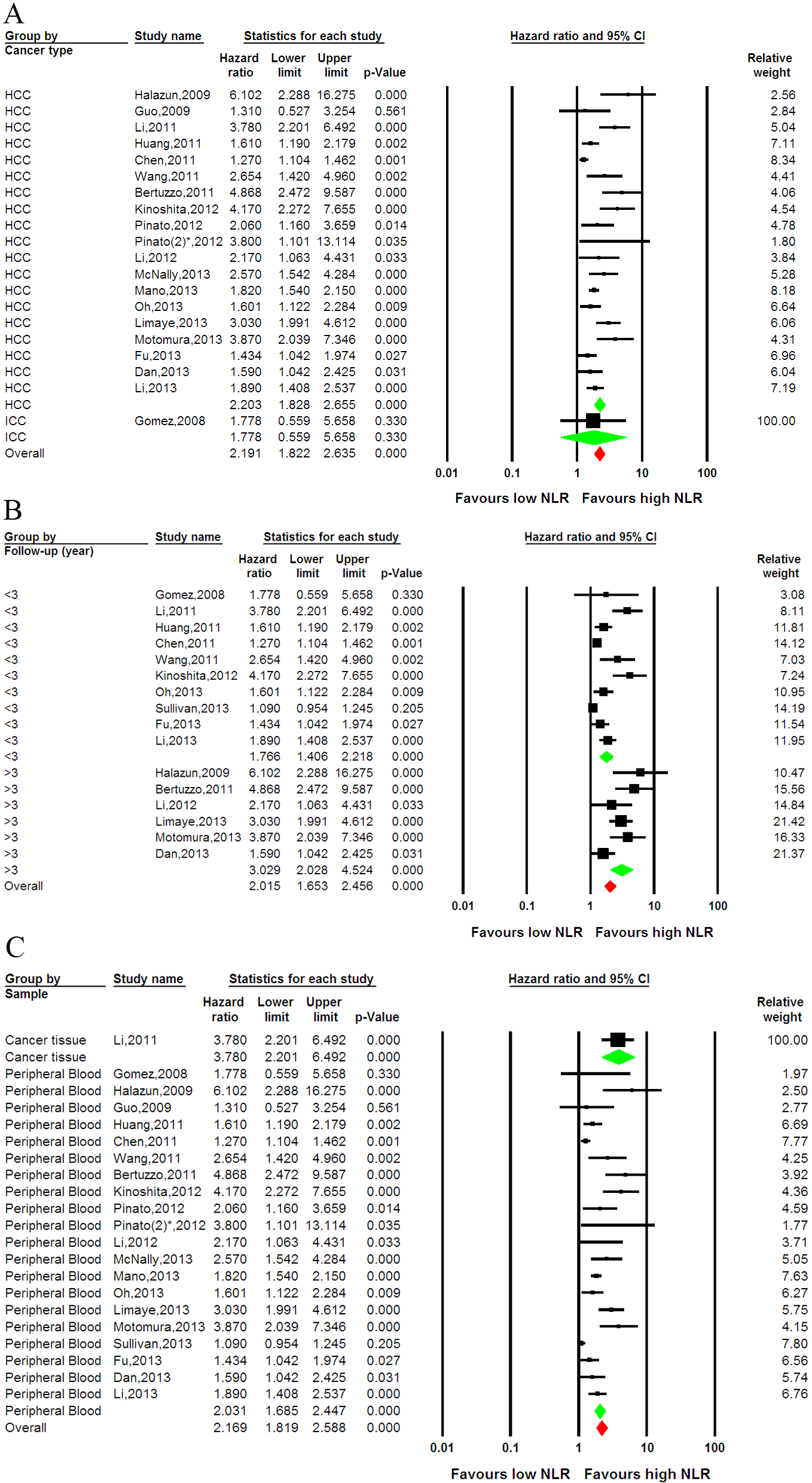

Supplement: Figure S3 — Stratified forest plots of the association between the NLR and OS. (A) Subgroup analysis was based on cancer type. (B) Subgroup analysis in studies with a median follow-up time less than or more than 3 years. (C) Subgroup analysis was based on the sampling method. Green represents the subgroup pooled effective size, whereas red represents the overall pooled effective size. NLR = neutrophil-to-lymphocyte ratio; OS = overall survival; CI = confidence interval; *, the different study by Pinato. (TIF) [file pone.0096072.s003.tif]

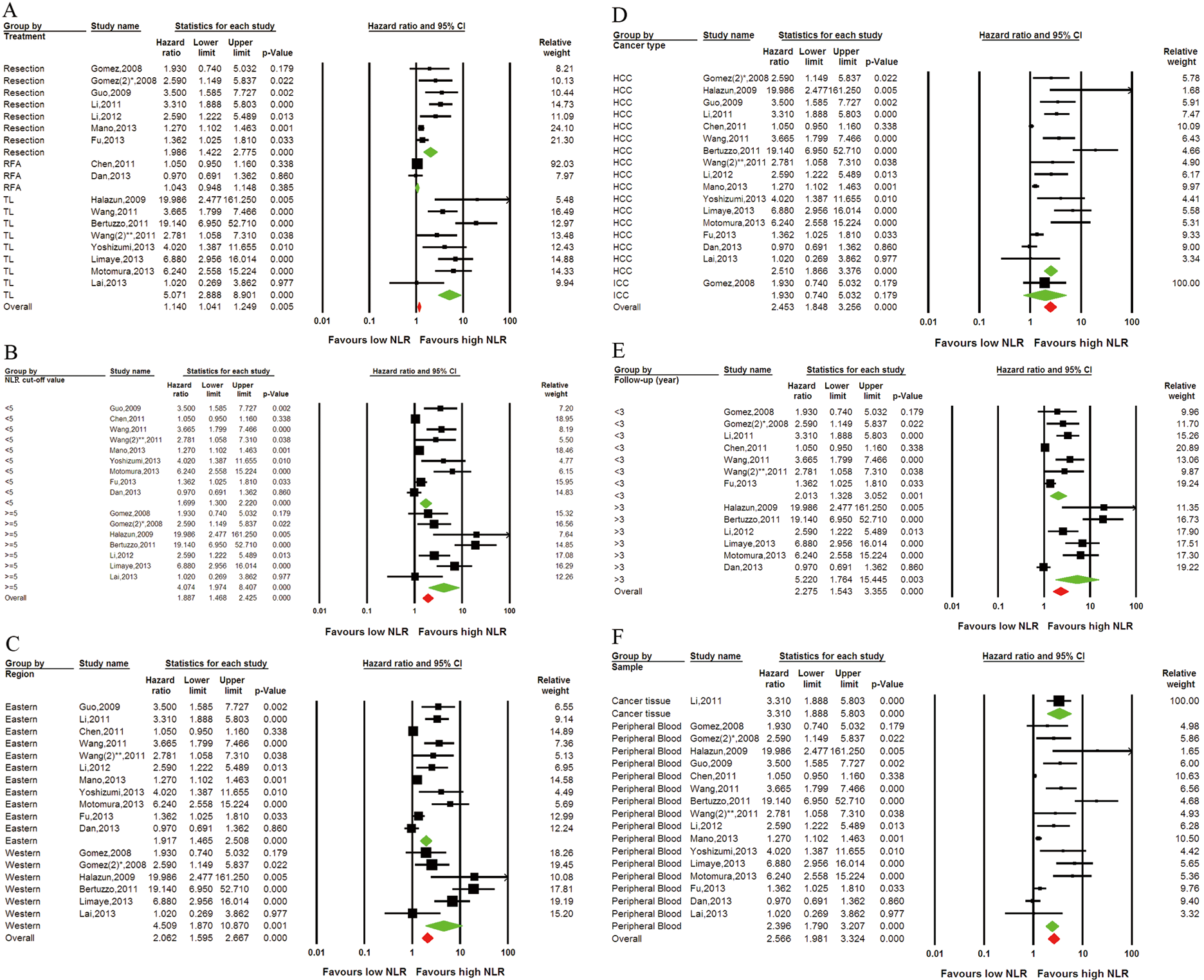

Supplement: Figure S4 — Stratified forest plots of the association between the NLR and DFS. (A) Subgroup analysis in patients who received different treatments. (B) Subgroup analysis in studies with an NLR cut-off value less than or greater than 5. (C) Subgroup analysis was based on the region in which the study was reported. (D) Subgroup analysis was based on cancer type. (E) Subgroup analysis in studies with a median follow-up time less than or greater than 3 years. (F) Subgroup analysis was based on the sampling method. Green represents the subgroup pooled effective size, whereas red represents the overall pooled effective size. NLR = neutrophil-to-lymphocyte ratio; DFS = disease-free survival; CI = confidence interval; *, the different study by Gomez; **, the different study by Wang. (TIF) [file pone.0096072.s004.tif]

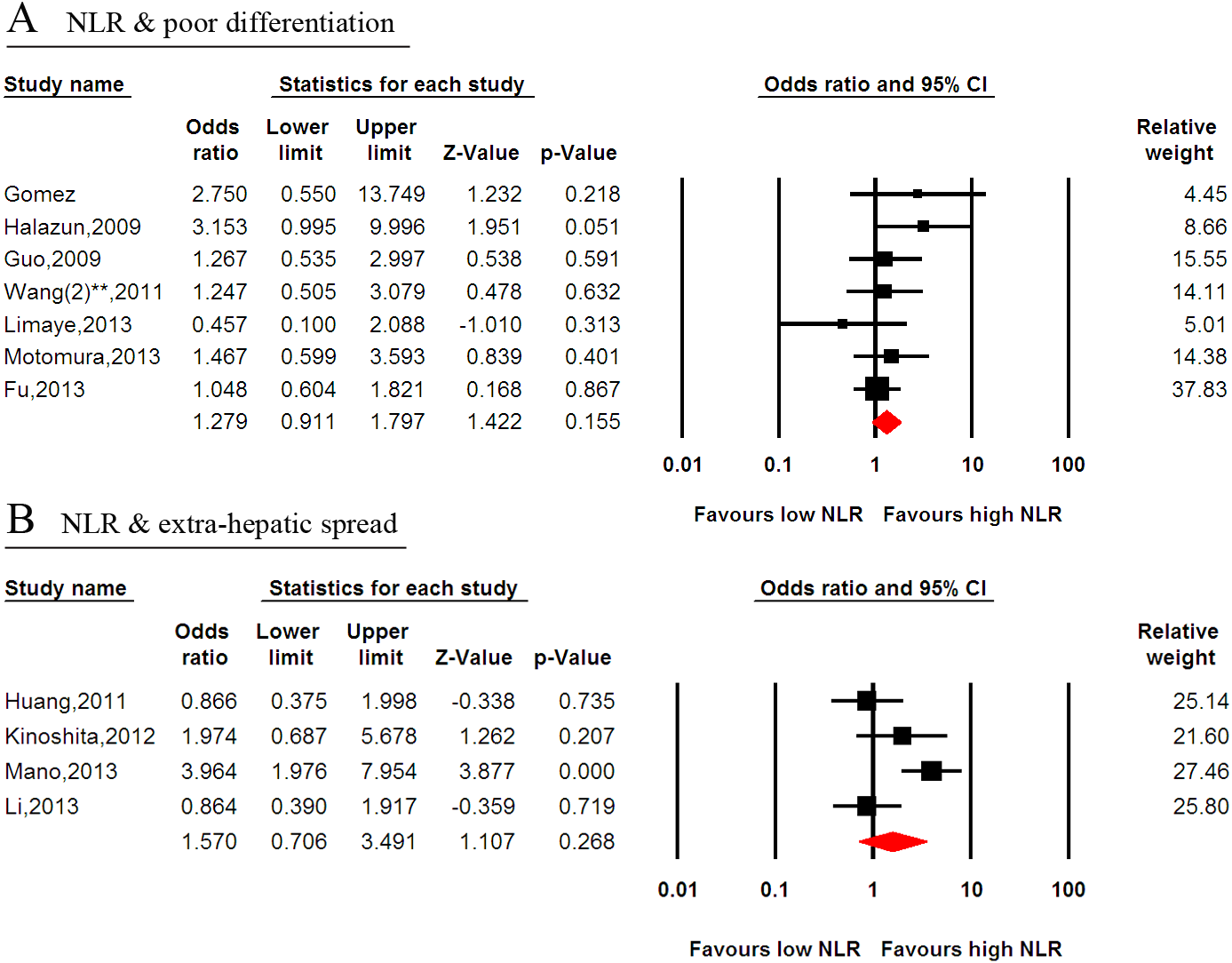

Supplement: Figure S5 — Forest plots of the association between the NLR and tumor characteristics. (A) The association between the NLR and differentiation of tumor cells. (B) The association between the NLR and extra-hepatic spread. Green represents the subgroup pooled effective size, whereas red represents the overall pooled effective size. NLR = neutrophil-to-lymphocyte ratio; CI = confidence interval; **, the different study by Wang. (TIF) [file pone.0096072.s005.tif]

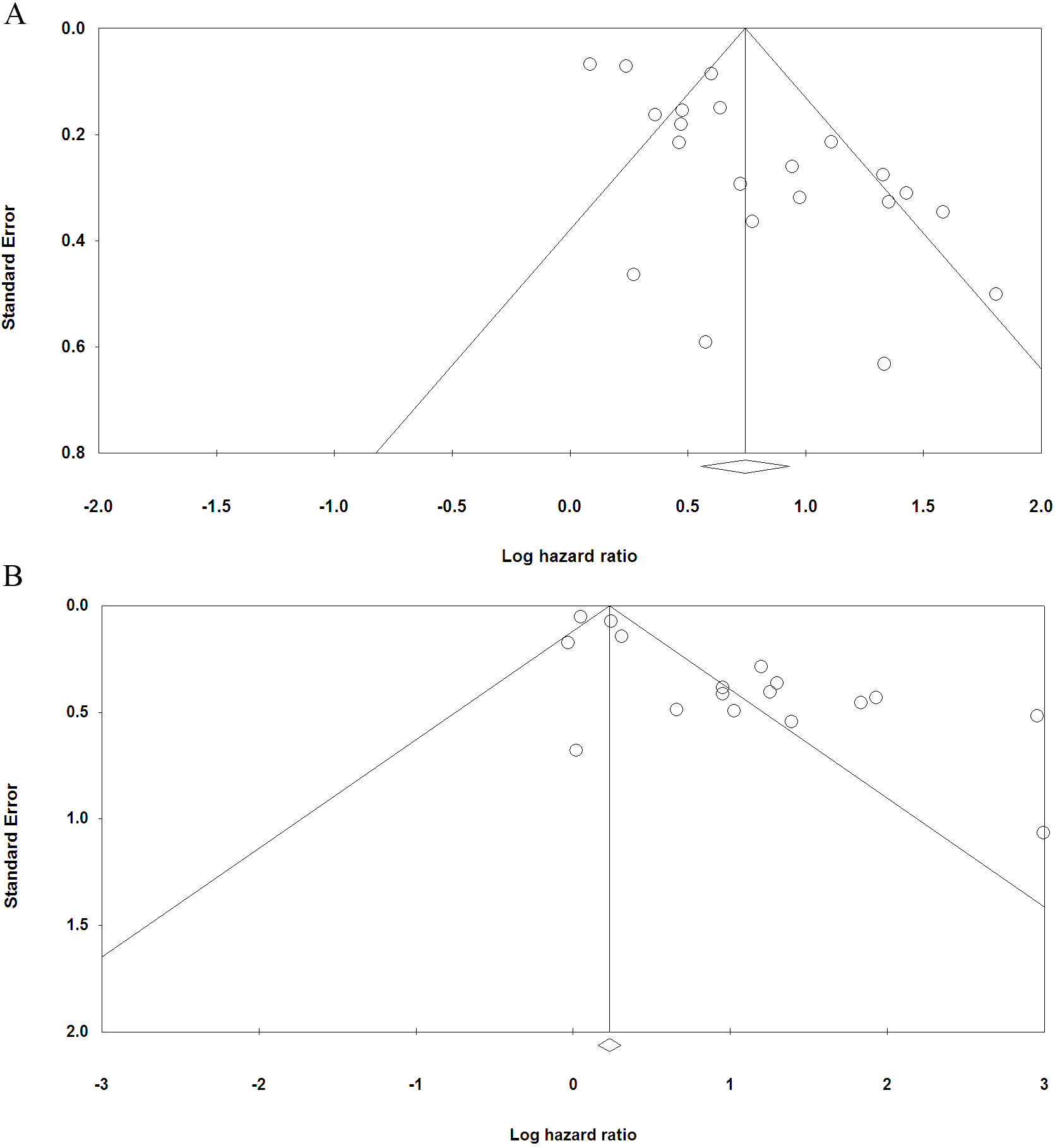

Supplement: Figure S6 — Funnel plots for the hazard ratios of recessive modes in the included studies. (A) OS (n = 26). (B) DFS (n = 17). (TIF) [file pone.0096072.s006.tif]
